# In-parallel resonators to increase the absorption of subwavelength acoustic absorbers in the mid-frequency range

**DOI:** 10.1038/s41598-019-47516-7

**Published:** 2019-07-31

**Authors:** Yves Aurégan, Maaz Farooqui

**Affiliations:** Laboratoire d’Acoustique de l’Université du Mans, Centre National de la Recherche Scientifique (CNRS), Le Mans Université, Avenue Olivier Messiaen, 72085 Le Mans, Cedex 9 France

**Keywords:** Applied physics, Acoustics, Engineering

## Abstract

The acoustic effects of in-parallel resonators is compared to the behavior of a classical single degree of freedom resonator for which the resistance and the mass are in series. In-parallel resonators serve to enhance the acoustic absorption for mid-frequencies thereby extending the active frequency range of perfect acoustic absorbers. Two implementations of these in-parallel absorbers are presented and investigated experimentally as well as numerically. In the first demonstration, the resistance is a perforated plate with a wiremesh and the oscillating mass is the air that fills a tube passing through the plate. The second implementation consists of a thin flexible beam that oscillates and where the resistance is due to the micro-slit resulting from the cutting of the beam.

## Introduction

There is an ongoing interest for sound absorbers with a low thickness to wavelength ratio, in order to reduce sound at low-frequencies with small size devices^1–3^. To achieve this goal, several metamaterial structures have been recently studied, like for instance space-coiling structures^4–8^, slow-sound materials^9–12^, Helmholtz resonators^13,14^ and membranes absorbers^1,15–17^. All these devices use acoustic resonances and it has been shown that, for a Single Degree Of Freedom (SDOF) resonator, the longer the wavelength relative to the size of the absorber, the narrower the operating frequency band. Thus, the problem of expanding the frequency band of sub-wavelength absorbers, studied intensively in 18, remains relevant and significant. To increase the effective frequency band, several resonators are often combined either in-series^12^, in-parallel or both^18^. The area actually dedicated to each frequency is then small and non-linear effects can occur on the active surfaces of each resonator when the amplitude of the incident wave is large. These non-linear effects can significantly modify the acoustic behavior of the material^19^.

In this paper, we present a different arrangement of the resonator elements that gives better absorption in the mid-frequencies range while being able to obtain perfect absorption at a moderate sub-wavelength ratio. We illustrate this effect with two implementations of these in-parallel absorbers. In the first demonstration, the resistance is a perforated plate with a wiremesh and the oscillating mass is the air that fills a tube passing through the plate. The second implementation consists of a thin flexible beam that oscillates and where the resistance is due to the micro-slit resulting from the cutting of the beam.

## Results

### In-parallel resonators

**Figure 1:**
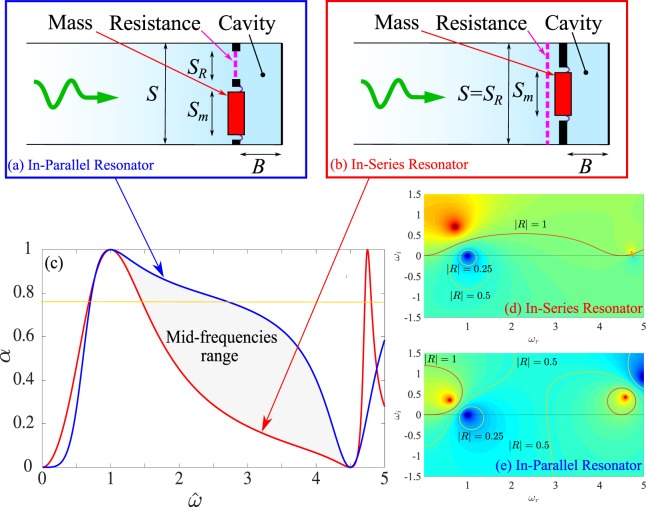
Sketch of SDOF resonators when the mass and the resistance are (**a**) in-series or (**b**) in-parallel. (**c**) Absorption coefficients (*α*) for perfect absorbers in-series and in-parallel for *C* = 2*π*/9 and (**d**,**e**) position of the poles (red) and zeros (blue) of the resonators in the complex *ω*-plane.

The SDOF structures can be studied in a simplified but informative way in the form of a system with only 3 components: mass, resistance and spring. The stiffness comes from the air compression in a cavity of area *S* and thickness *B*. The two sub-systems, namely the moving mass *m* acting on an area *S*_*m*_ and the resistance *R*_*e*_ acting on an area *S*_*R*_, can be placed in-series or in-parallel, as shown in Fig. 1(a,b).

The absorbers are characterized by their normalized impedance *Z* = *p*_*i*_/*Z*_0_*u*_*i*_ where *p*_*i*_ and *u*_*i*_ are the pressure and velocity of the incident wave and *Z*_0_ = *ρ*_0_*c*_0_ is the characteristic impedance of air, where *c*_0_ is the sound velocity and *ρ*_0_ is the air density. To simplify the comparison between the different absorbers, we normalize the frequency with a target frequency *f*_*R*_ such as $\hat{\omega }=\omega /2\pi {f}_{R}$. Each of the three components can itself be described by an impedance. The cavity impedance, linked to the compressibility of air, can be written ${Z}_{c}={p}_{c}/{Z}_{0}{v}_{c}={({\rm{i}}{\rm{t}}{\rm{a}}{\rm{n}}(\hat{\omega }C))}^{-1}$ where *p*_*c*_ is the acoustic pressure in the cavity and *C* = *ω*_*R*_*B*/*c*_0_ = 2*πB*/*λ*_*R*_ is inversely proportional to the sub-wavelength ratio. *λ*_*R*_ is the wavelength at the target frequency and the convention e^i*ωt*^ has been chosen. The mass impedance is ${Z}_{m}={\rm{d}}{p}_{m}/{Z}_{0}{v}_{i}={\rm{i}}\hat{\omega }L$ where $L={\omega }_{R}mS/({S}_{m}^{2}{\rho }_{0}{c}_{0})$ and d*p*_*m*_ is the pressure difference between the two faces of the mass. The impedance of resistance is *Z*_*R*_ = d*p*_*R*_/*Z*_0_*v*_*i*_ = *R* where d*p*_*R*_ is the pressure difference between the two faces of the resistance and *R* = *R*_*e*_*S*/*S*_*R*_.

If the resistance and the mass are in-series the absorber impedance is ${Z}_{{\rm{I}}{\rm{S}}{\rm{R}}}=R+{\rm{i}}\hat{\omega }L+({\rm{i}}\,{\rm{t}}{\rm{a}}{\rm{n}}(\hat{\omega }C){)}^{-1}$, as described in^13^. If, on the other hand, the resistance and the mass are in-parallel, the impedance of the absorber is

1 $${Z}_{{\rm{IPR}}}=\frac{1}{1/R+1/i\hat{\omega }L}+\frac{1}{{\rm{i}}\,\tan (\hat{\omega }C)}.$$

To obtain perfect absorption for the target frequency ($\hat{\omega }=1$) in the case of an In-Series Resonator (ISR), it is necessary to have *R* = 1 and *L* = 1/tan(*C*). The curve giving the absorption of such a SDOF in-series resonator is given in Fig. 1(c) by the red curve in the case where the sub-wavelength ratio is equal to 9. For an In-Parallel Resonator (IPR), the mass and the resistance leading to perfect absorption at $\hat{\omega }=1$ are given by

2 $$L=\frac{1+\,\tan \,{(C)}^{2}}{\tan (C)}\,{\rm{and}}\,R=\frac{1+\,\tan \,{(C)}^{2}}{\tan \,{(C)}^{2}}.$$

The absorption coefficient *α* of a perfect absorber IPR is given in Fig. 1(c) by the blue line for the same sub-wavelength ratio as the ISR.

The interest of using an IPR versus a classical resonator where the elements are in-series appears clearly in the mid-frequencies range ($\hat{\omega } > 1.5$ and $\hat{\omega }$ smaller than the half-wave length resonance of the cavity) where the absorption is increased. In the particular case given in Fig. 1(c), the absorption increases from *α*_ISR_ = 0.19 to *α*_IPR_ = 0.72 at $\hat{\omega }=3$. This increase in mid-frequencies is compensated by a slight decrease in absorption in the very low frequency ($\hat{\omega } < 0.5$) to fulfill the causality constraint on absorption given in^18^. This effect is illustrated in Fig. 1(d,e) where the color map of the modulus of the reflection coefficient is plotted when the frequency is extended in the complex plane by $\hat{\omega }={\omega }_{r}+{\rm{i}}{\omega }_{i}$. By construction of a perfect absorber, the zero (deep blue) of the reflection coefficient is on the real axis at $\hat{\omega }=1$. The effect of the IPR is to move the pole (deep red) closer to the real axis and at a lower real frequency. The lines for which the reflection coefficient is 0.5 (*α* = 0.75, in yellow on Fig. 1(d,e)) are then substantially modified compared to ISR case.

The mechanism that improves the behavior in the mid-frequency range is the short-circuiting of the mass. Indeed, in the ISR case, the increase in the inertial effects is proportional to the frequency, and it shifts the impedance of the absorber away from the optimal impedance when the frequency exceeds the resonance frequency. The increase in the reactance, ℑ(*Z*_*IPR*_), due to the mass is cancelled out at “high” frequencies when it is placed in parallel to the resistance. In fact, at high frequencies (but when $\tan (\hat{\omega }C)$ can be considered equal to $\hat{\omega }C$), the impedance of the absorber can be approximated by ${Z}_{{\rm{ISR}}}=R+{\rm{i}}\hat{\omega }L+O(1/\hat{\omega })$ when the elements are connected in series and by ${Z}_{{\rm{IPR}}}=R+O(1/\hat{\omega })$ when they are connected in parallel.

**Figure 2:**
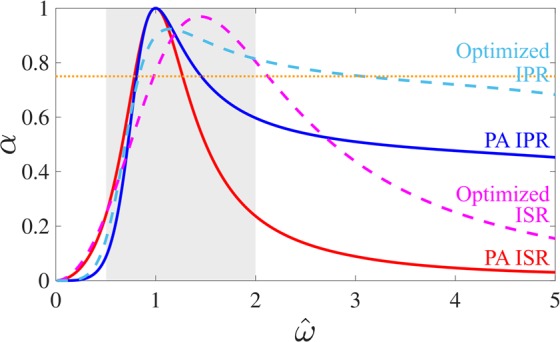
Absorption coefficients (*α*) of the in-series and in-parallel resonator when the perfect absorption is achieved for *C* = 2*π*/15 (continuous line). Absorption coefficients of the optimized in-series and in-parallel resonators for *C* = 2*π*/15 (dashed lines). Optimization maximizes the average value of *α* for $0.5 < \hat{\omega } < 2$ (in grey on the figure).

When the constraint of having a perfect absorption for $\hat{\omega }=1$ is released, the effective frequency band of the resonators can be further extended as shown in the Fig. 2. The solid lines represent the absorption of in-series and in-parallel resonators for a sub-wavelength ratio of 15 when the perfect absorption is achieved. For *C* = 2*π*/15, the resistance *R* and inductance *L* that lead to perfect absorption are *R*_*ISR*_ = 1 and *L*_*ISR*_ = 2.25 in the ISR case and *R*_*IPR*_ = 6.04 and *L*_*IPR*_ = 2.69 in the IPR case. To expand the effective frequency band, the average absorption coefficient *α*_*m*_ between $0.5 < \hat{\omega } < 2$ (average over 1000 values in the frequency range) was computed for different values of *R* and *L*. The ISR and IPR optimum absorption coefficient *α*, plotted as dashed lines on the Fig. 2, corresponds to the values of *L* and *R* which maximizes *α*_*m*_ (*R*_*ISR*_ = 1.40, *L*_*ISR*_ = 0.99 and *R*_*IPR*_ = 3.07, *L*_*IPR*_ = 2.79). In this case, perfect absorption is no longer achieved but the frequency range where the absorber is efficient is wider.

### Experimental implementations

Several practical implementations of this in-parallel resonator idea are possible. The oscillating mass can be made, for example, with air moving in a neck, with a plate glued to a membrane or with the use of a deformable element. The resistance can be achieved by means of a porous sheet, a perforated or micro-perforated plate, a resistive mesh or by means of micro-slits.

#### In-parallel Helmholtz resonators

**Figure 3:**
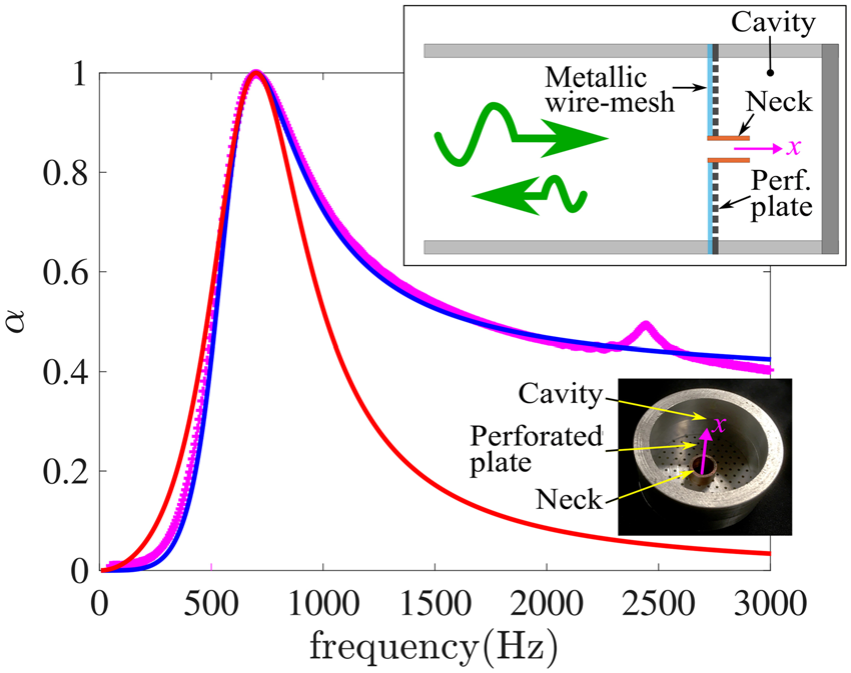
Measured absorption coefficient *(α*) of an in-parallel absorber (magenta symbols) and the absorption predicted using Eq. 1 with *C* = 2*π*/16.4, *L* = 2.9 and *R* = 7.1 (blue line). The red line is the absorption of the ISR with the same *C*. The inset is a picture of the measured IPR where the bottom of the cavity has been removed for clarity.

A very simple realization of an IPR is given in the inset of Fig. 3. It consists of a perforated plate on which a metallic wire-mesh has been glued to increase the resistance. This plate has been drilled in its center to insert a tube that acts as a neck in which an air mass will oscillate. This implementation is very close to a Helmholtz resonator except that the dissipation does not occur in the neck but instead in the resistive plate which makes the incident wave communicate directly with the cavity. In our measured device, the tube has an inner diameter of 6 mm, an outer diameter of 8 mm and a length of 7 mm. The cavity has an inner diameter of 30 mm and a total length *B* = 30 mm. The cavity is closed by a Plexiglas disk. The normalized resistance of the plate is measured as *R*_*e*_ =  7.3.

The targeted frequency of this IPR was 700 Hz and, with a 3 cm cavity, it leads to a sub-wavelength ratio of 16.4. Thus, to have a perfect absorber the Eq. 2 lead to the theoretical values *L* = 2.9 and *R* = 7.1. The experimental values were *L* = *ω*_*R*_*HS*/(*S*_*m*_*c*_0_) = 3.38 and *R* = *R*_*e*_*S*/*S*_*R*_ = 7.90 where *H* is the effective length of the neck. The predicted absorption coefficient using Eq. 1 is in very good agreement with the measured absorption coefficient as shown in Fig. 3. The most noticeable difference is a small peak in experimental results around 2450 Hz. One possible reason behind this peak is the vibration of the perforated plate caused by the pressure difference between the cavity and the measuring tube.

#### Beam with micro-slits

**Figure 4:**
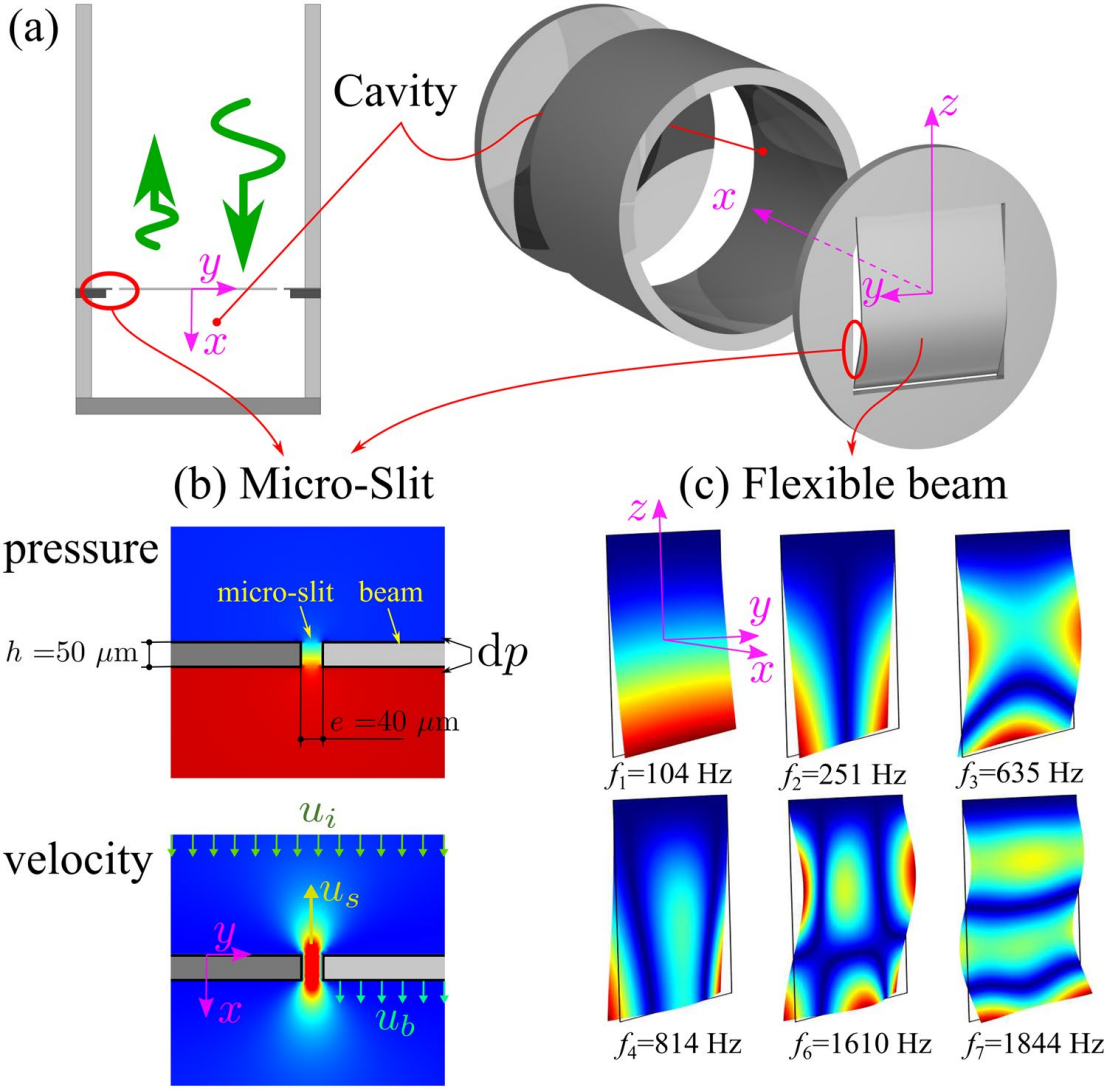
(**a**) Sketch of the beam with micro-slit resonator. (**b**) Pressure and vertical velocity around the micro-slit computed at *f* =  1000 Hz allowing for thermo-viscous interactions. (**c**) Some of the mode shapes of the thin titanium beam.

**Figure 5:**
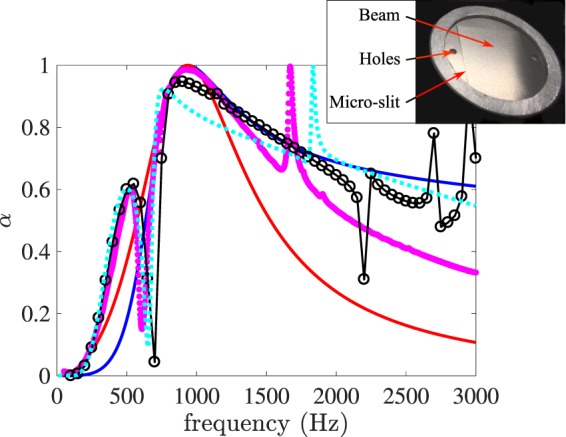
Measured absorption coefficient of a beam in-parallel absorber (magenta symbols). The blue and red lines are respectively the absorption coefficients of the IPR and ISR with perfect absorption for *C* = 2*π*/12. The dotted cyan line corresponds to the lumped element model and the black circles corresponds to the COMSOL computation.

A second realization of an IPR, depicted in Fig. 4(a) and the inset of Fig. 5, uses a deformable plate over a cavity of thickness *B* = 30 mm. To meet the perfect absorption conditions, this plate of surface 20 × 20 mm^2^ must be very flexible and lightweight, so that we chose to cut it from a 50 *μ*m plate of titanium. Due to its very high flexibility, this titanium plate was glued on a more rigid plate in which a cutout (21 × 21 mm^2^) a little larger than the beam was made. The mode shape of some of the first in-vacuo modes of the plate are plotted in Fig. 4(c). Some of these modes are torsional modes (see for instance the second mode at *f*_2_ = 251 Hz). For a symmetry reason, the average velocity over this mode vanishes and this mode does not affect the acoustic response of the resonator. The only important modes are those that have an almost one-dimensional behavior (mode 1, 3, 7, …). Thus, the behavior of the plate can be very reasonably approximated by calculating the dynamic deformation of a beam subjected to a uniform loading. The ratio between pressure and average plate velocity is the plate impedance *Z*_*p*_, which can then be computed simply with one-dimensional finite differences method.

To obtain a large pressure difference d*p* between the two sides of the beam that is necessary to set it in motion, the U-shaped slit all around the beam must be very thin. This has been done by using laser micro-cutting that allows a width of the slit no larger than 40 *μ*m. It can be seen from the numerical results of Fig. 4(b) that the pressure, in the vicinity of the beam, only varies in the slit and that it is a reasonable approximation to consider the pressure as uniform on both sides of the beam and of the slit. The sound propagation in a thin slit of small height is described by its transfer impedance *Z*_*s*_ given by^20^:

3 $${Z}_{s}=\frac{{\rm{d}}p}{{Z}_{0}{v}_{s}}=\frac{{\rm{i}}\omega h}{{c}_{0}}\frac{1}{1-\,\tanh (\alpha e/2)/(\alpha e/2)},$$

where *v*_*s*_ is the averaged velocity in the slit, *e* and *h* are the thickness and the height of the slit and $\alpha =\sqrt{{\rm{i}}\omega {\rho }_{0}/\mu }=$ $(1+{\rm{i}})/{\delta }_{a}$ where *μ* is the dynamic viscosity and *δ*_*a*_ is the viscous boundary layer thickness. This boundary layer thickness varies from *δ*_*a*_ = 100 *μ*m at 500 Hz to *δ*_*a*_ = 40 *μ*m at 3 kHz. In this frequency band, the flow in the slit of 40 *μ*m can therefore be approximated by an incompressible oscillating Poiseuille flow that can be used to approximate (Eq. 3) by *Z*_*s*_ = 12 *μh*/(*ρ*_0_*c*_0_*e*^2^) + i*ω*6*h*/5*c*_0_. The slit has not only a resistive effect but also an inertial effect due to the oscillation of the air in the slit. We can see in Fig. 4(b) that the oscillating air mass extends outside the slit and that an added length must be included to the height that appears in the imaginary part of *Z*_*s*_. This added length, which has long been known for wide pipes^21^, is less well known for slits with strong viscous effects. In such cases, the length correction is very dependent on the geometry and exact acoustic conditions^22^. In the Eq. 3, we used an effective slit height *h*_eff_ = 6*h*/5 + 1.6*e* that approximates our numerical calculations (as described in the next paragraph) and the values available in the literature^23,24^. To obtain a lumped element model of the beam with micro-slit resonator, we use Eq. 1 where *R* is substituted by *SZ*_*s*_/*S*_*s*_, *S*_*s*_ is the surface of the slot (0.04 × 60 mm^2^) and where ${\rm{i}}\hat{\omega }L$ is substituted by *SZ*_*p*_/*S*_*p*_, *S*_*p*_ is the surface of the plate (20 × 20 mm^2^). The results of this model are plotted in cyan dotted lines with the experimental results (magenta symbols) on the Fig. 5. The agreement between the model and the experiment is good for low frequencies but less satisfactory around the frequency for which the experimental device has perfect absorption, as well as for high frequencies.

To obtain a better agreement, a three-dimensional numerical simulation of the system was carried out using COMSOL Multiphysics. The simulation of the plate vibrations was performed using the module *Solid Mechanics*, the acoustics in the slit and in its surroundings was calculated using the module *Thermoacoustics* while the incident acoustic propagation in the cavity was calculated using the module *Acoustics*. The appropriated interface conditions were applied at the boundaries between the three computation domains. The results are shown by black circles on the Fig. 5. The results compare better than the lumped model to the experimental results in the vicinity of perfect absorption but also differ significantly from the experiment for the highest frequencies. A possible reason for the inaccurate high-frequency peaks is the incomplete consideration in the numerical calculations of the details of the vibrating plate, which is actually composed of 2 plates glued one on top of the other.

## Discussion

The use of a in-parallel resonator significantly increases the absorption efficiency in the mid-frequency range compared to more conventional in-series resonators. This kind of device can have a large open area to minimize non-linear effects. An important effect of the in-parallel resonators is that their reactances ℑ(*Z*_*IPR*_) tends to zero as the frequency increases. This means that the impedance tends to a purely real value, which is interesting for some applications such as acoustic attenuation in aircraft nacelles^25^. The use of a flexible plate with micro-slits allows perfect sound absorption and also allows energy harvesting to be considered^17,26^.

## Methods

### Experimental setup

The reflection coefficients of the in-parallel resonators are measured in a homemade impedance tube. It consists of a hard walled steel duct (diameter 30 mm) where four microphones (B&K 4938 microphones with Nexus 2690 amplifiers) are mounted. The distances between the microphones and the one closest to the sample are respectively 30, 130 and 414.6 mm. The use of four microphones allows an over-determination of the measurement of the ratio between the reflected wave and the incident wave in order to have a good accuracy over the entire frequency range. The incident wave is produced by a source composed of two loudspeakers acting respectively in low frequency (<700 Hz) and high frequency (>500 Hz) ranges. Then, the reflection coefficient has been accurately measured from 50 to 3000 Hz with steps of 2 Hz. At each change of frequency, a settling time of 500 cycles is used to establish the response and the measurement is made over a duration of 1000 cycles.
